# Epidemiological Chronicle of the First Recovered Coronavirus Disease Patient From Panama: Evidence of Early Cluster Transmission in a High School of Panama City

**DOI:** 10.3389/fpubh.2020.553730

**Published:** 2020-09-15

**Authors:** Augusto Hernandez, Paul Muñoz, Jose C. Rojas, Gilberto A. Eskildsen, Julio Sandoval, K. S. Rao, Rolando A. Gittens, Jose R. Loaiza

**Affiliations:** ^1^Unidad de Cuidados Intensivos, Complejo Hospitalario Doctor Arnulfo Arias Madrid, Caja de Seguro Social (CSS), Panama City, Panama; ^2^Colegio Monseñor Francisco Beckmann, Panama City, Panama; ^3^Centro de Biodiversidad y Descubrimiento de Drogas, Instituto de Investigaciones Científicas y Servicios de Alta Tecnología, Panama City, Panama; ^4^Unidad de Cuidados Intensivos, Ministerio de Salud de Panamá (MINSA), Panama City, Panama; ^5^Smithsonian Tropical Research Institute, Panama City, Panama; ^6^Programa Centroamericano de Maestría en Entomología, Universidad de Panamá, Panama City, Panama

**Keywords:** epidemiological investigation, SARS-CoV-2, COVID-19, anosmia, dysgeusia, Panama City

## Abstract

The first patient infected by severe acute respiratory syndrome coronavirus 2 (SARS-CoV-2) in Panama was reported on March 9, 2020. Here, we describe the first case of recovery from coronavirus disease 2019 (COVID-19) in the country. The patient was a 49-year-old male high school teacher, who did not show any primary symptoms of COVID-19 described by health authorities as the signs for medical attention. Nonetheless, he became severely ill over the course of 2 weeks and almost lost the battle against COVID-19. The identification of the first cluster of SARS-CoV-2 community transmission in the secondary school where the patient of this case report taught, led to the closure of the school and, a day after, the shutdown of the national education system, which may have prevented the spread and slowed the transmission rate of COVID-19 during the early stages of invasion. This case report highlights the need to increase awareness among healthcare professionals in Latin America to consider symptoms such as anosmia and dysgeusia as the sentinel signs of COVID-19 infection in order to prevent deaths, especially in high-risk patients.

## Introduction

Severe acute respiratory syndrome coronavirus 2 (SARS-CoV-2) is the etiological agent of the coronavirus disease (COVID-19) pandemic that has affected more than 100 countries, with more than 19.3 million confirmed cases (1). High fever (38°C), dry cough, and shortness of breath are the most common symptoms resulting in patients requiring oxygen treatment, and some needing immediate access to the intensive care unit (ICU) due to respiratory distress with ≥50% probability of death (2). Globally, a lethality rate ranging from 2.1% (South Korea) to 14.3% (Italy) has been reported, depending on case surveillance strategy and number of tests (per million people) across countries affected by COVID-19 (1–3). The infection severely affects people >60 years of age, while children and young adults are often oligosymptomatic. Nonetheless, the infection could be dangerous in younger individuals with underlying diseases (2, 3). With no specific antiviral drug therapy or an effective vaccine against SARS-CoV-2 in the near future, patients under critical conditions are treated with the standard supportive care practices for acute respiratory distress syndrome (4). At the same time, public health outbreak response measures are based on enforcing isolation, quarantine, and social distancing to mitigate the spread of the disease, and reduce the number of people requiring hospitalization (3–5).

Panama City is the second most populous urban center in Central America and a hub of international trade and tourism. It has a metropolitan population of 1.6 million people, and ~2.5 million visitors arriving from abroad annually (6). The Ministry of Health in Panama (MINSA, for its initials in Spanish), with the support of the Pan-American Health Organization, had established a strong containment strategy that covered all ports of entry into the country since March 16, 2020. Extra stringent actions of community containment such as closing schools and universities, private sector companies, and government offices were also implemented by March 12, 2020, including limiting large gatherings of people in commercial centers, sport arenas, and other public spaces (7). The main goal of these actions was to reduce the transmission rate of the virus for 14–20 days after a cluster of activity had been identified, and protect the overall healthcare system capacity.

Currently, attempts to prevent the spread of the virus are no longer focused only on the close contacts of confirmed cases within familial households or work-related spaces. Strict social distancing measures are being implemented to help decrease the spread of the virus, but the curve has not flattened yet. As of August 7, 2020, SARS-CoV-2 has infected 71,418 people in the country, and 1,574 have died (1, 8). Although it is believed that the initial infections in Panama originated from travelers who entered the country from Europe and the US (9, 10), the epidemiological scenario surrounding a potential index case has not yet been established in the country. In this report, we describe the epidemiological chronicle of the first COVID-19 recovery case in Panama. This patient is a high school teacher in Panama City, who initially had no fever or dry cough, but instead presented loss of appetite, anosmia, and dysgeusia along with episodes of mild-to-moderate dyspnea that worsened over 2 weeks.

## Case Report

**Figure 1:**
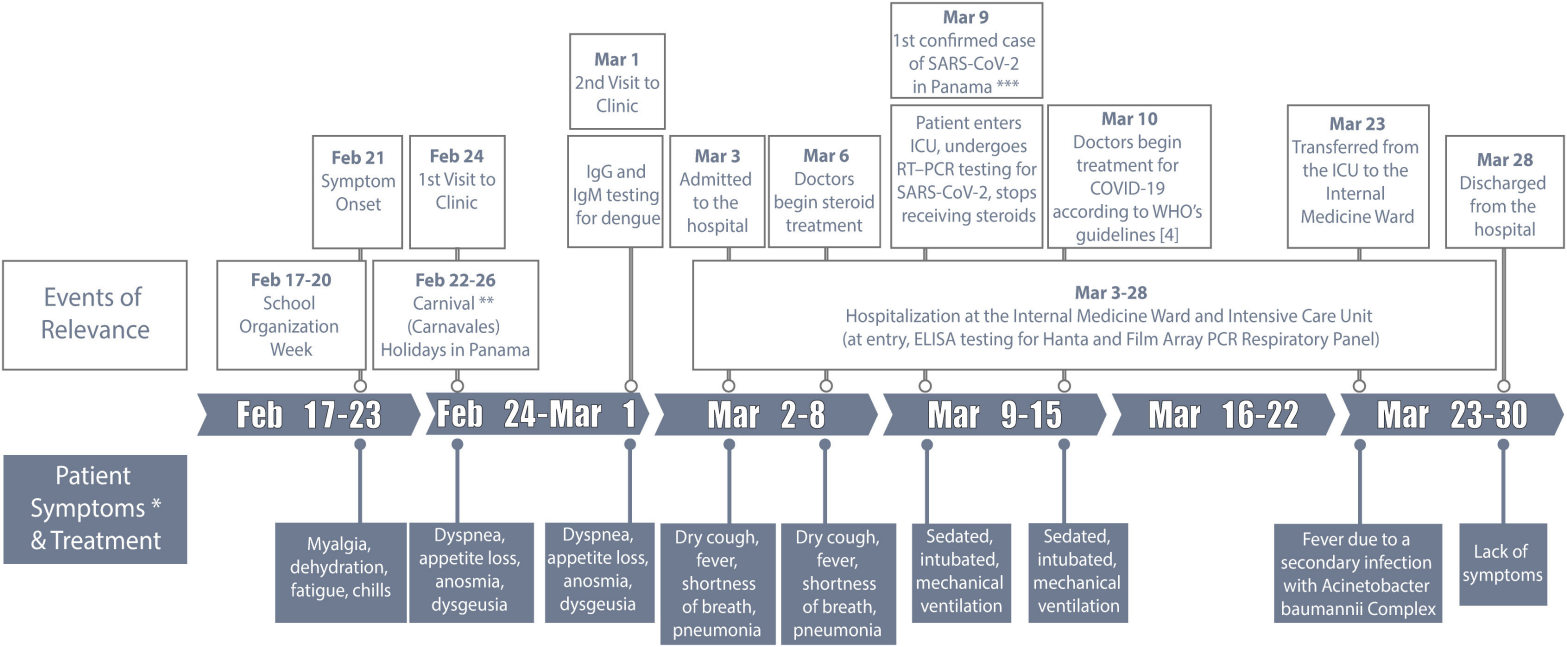
Case history of the first recovered coronavirus disease (COVID-19) patient from Panama. *The patient experienced no fever, sore throat, nasal congestion, running nose or severe coughing since symptom onset until the day of hospitalization. **Carnivals (“Carnavales”) are one of the most popular holiday celebrations of the year in Panama. ***The first confirmed case of severe acute respiratory syndrome coronavirus 2 (SARS-CoV-2) in Panama using reverse-transcription polymerase-chain-reaction (RT-PCR) testing was reported on March 9, 2020, and the same day the patient was confirmed positive for SARS-CoV-2 infection. The school where the first cluster of activity was detected in Panama City closed on March 10, 2020, 5 days after the beginning of the academic year. ICU, Intensive care unit; IgG, Immunoglobulin G.

Written and signed informed consent was obtained from the patient to publish this case report. On February 21, 2020, a 49-year-old male with flu-like symptoms such as myalgia, dehydration, fatigue, and chills, but without fever, cough, or respiratory distress visited the hospital to seek medical treatment. He was sent back home with prescriptions by the medical doctor for a probable viral infection. At that point, there were no reported cases of COVID-19 in Panama. Prior to this time, the patient was healthy, without any preexisting medical conditions and had not traveled outside Panama in the last 12 months. As the symptoms continued, including additional ones such as diarrhea, dizziness, and dyspnea, the patient visited a private clinic twice in <6 days, and tested negative for dengue virus (Figure 1). A week after symptom onset, his body temperature (37.0°C, 98.6°F) and total white blood cell count (9.2 × 103 cells/μl) were in the normal range, but he would easily get tired after simple activities such as walking up the stairs of the school. During the first days of the academic year in Panama (March 1–3, 2020), he was unable to smell (anosmia) or taste any food (dysgeusia). He had diminished appetite but decided to continue teaching biology to ~40 teenage students.

**Figure 2:**
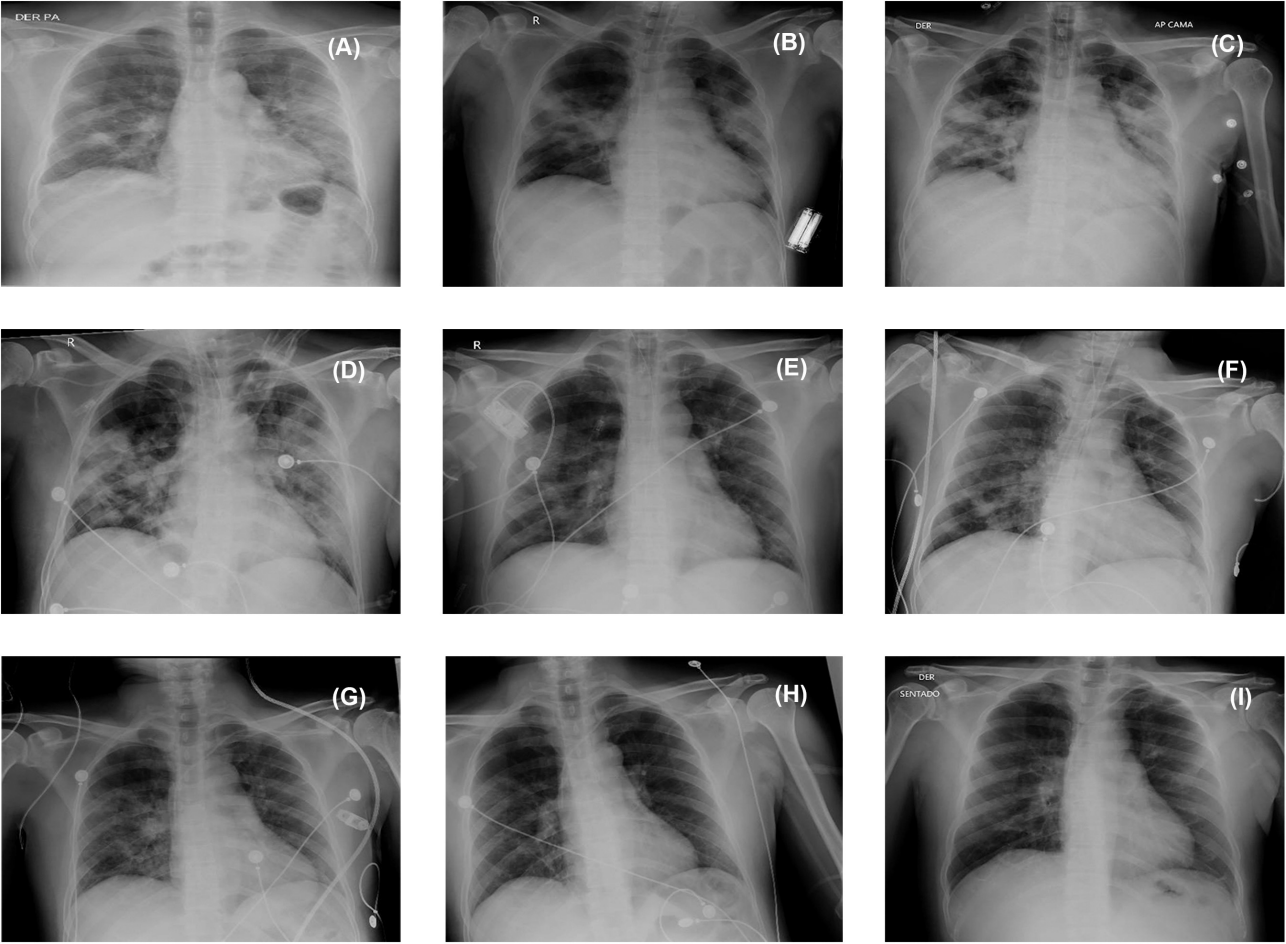
Chest radiographs. The radiographs show bilateral ground-glass opacities **(A)** on admission to the hospital on March 3, 2020; **(B)** during intubation on March 5; **(C)** during begin treatment with steroids on March 6; **(D)** on entering the intensive care unit on March 9; **(E)** on March 12; **(F)** on March 17; and **(G)** on March 20. No alveolar infiltrates observed on the radiographs **(H)** on March 23 and **(I)** during discharge from intensive care unit on March 24, 2020.

On March 3, 2020, he was admitted to the public hospital Caja de Seguro Social (CSS) Complejo Hospitalario Doctor Arnulfo Arias Madrid, along with the 64-year old male director of the same school, who had high fever, severe cough, and persistent respiratory problems. It is important to note that this occurred 6 days before MINSA officially confirmed the first case of COVID-19 in Panama in a Panamanian citizen who had recently returned from Spain on March 8, 2020. On arrival, the high school teacher's symptoms had evolved to dry cough, fever, and shortness of breath (Figure 1), and his chest radiograph showed extensive areas of multilobar opacities and bilateral minor pleural effusions, suggesting pneumonia (Figures 2A–D) (11). At that point, the complete blood count (CBC) showed leukocytosis (82.1% neutrophils) and hyperglycemia (Table 1). These measurements exceeded or were short of the normal range from day 2 until day 18 of hospitalization. In addition, CBC from day 8 to day 21 showed low levels of hemoglobin and hematocrit (Table 1). Moreover, on March 3, 2020, he was tested for a panel of respiratory diseases/pathogens by the Film Array polymerase chain reaction (PCR) technique (BioFire; Salt Lake City, USA), including several strains of coronavirus known to infect humans, and other numerous viral and bacterial pathogens. However, all results were categorized as undetected (Table 1). Additional laboratory testing was completed on day 2 of hospitalization, including microbiological culture, urinalysis, and serum chemistry. Between day 1 and day 18, the patient presented leukocytosis with neutrophilia, lymphopenia, and hypoalbuminemia (Table 1). From day 2–21, there were some alterations in the hepatic function, including higher levels of alanine transferase (ALT), aspartate transferase (AST), and lactate dehydrogenase (LDH) (Table 1). The chest radiographs continued to show signs of bilateral alveolar infiltrates until day 20, but signs of improvement were seen from March 12 (Figures 2E–G). Despite not knowing the cause of the patient's illness initially, on March 6, 2020, the medical team decided to initiate treatment with cephalosporins and continuous infusion of methylprednisolone (100 mg/24 h) for 4 days (Figure 1).

**Table 1:** Clinical results of the first recovered coronavirus disease (COVID-19) patient from Panama.

**Measure**	**Reference range**	**Day 1**	**Day 2**	**Day 8**	**Day 10 ICU**	**Day 16 ICU**	**Day 18 ICU**	**Day 21**
		**Mar 3**	**Mar 4**	**Mar 10**	**Mar 12**	**Mar 18**	**Mar 20**	**Mar 23**
White cell count (/μl)	3.9–11.5 × 10^3^	13.0	15.3	17.5	16.8	25.5	14.7	7.6
% Neutrophil	50–70	82.1	88	86.2	89.9	91.5	81	59.4
Platelet count (/μl)	150–400	281	249	464	515	455	342	410
% Lymphocytes	25–50	8.5	6.2	6.5	6.7	2.7	10.2	22.2
Red blood cell count (per μl)	4.0–6.2 × 10^6^	4.51	4.0	3.57	3.37	3.32	3.74	3.8
Hemoglobin (g/dl)	12.5–18	12.7	12.5	11.3	10.5	9.9	11.2	11.5
Hematocrit (%)	36–50	41.9	37.7	35	32.2	32.3	35.4	35.8
Glucose (mg/dl)	70–105	126	151	119	115	178	77	80
Blood urea nitrogen (mg/dl)	6–20	11	12	18	21	35	22	25
Creatinine (mg/dl)	0.7–1.2	1.07	0.85	0.80	0.65	0.59	0.60	0.60
Albumin (g/dl)	3.4–4.8	-	2.8	2.7	2.6	3.1	2.9	3.4
Sodium (mEq/liter)	136–145	137	136	146	145	145	138	140
Potassium (mEq/liter)	3.5–5.1	4.3	3.9	4.5	4.0	4.4	3.1	4.7
Chloride (mEq/L)	98–107	97	100	103	103	106	100	104
Alanine transferase (U/L)	10–50	-	146	150	282	530	241	133
Aspartate transferase (U/L)	10–38	-	178	107	252	146	50	48
Lactate dehydrogenase (U/L)	120–230	-	372	424	605	414	335	356
Creatine phosphokinase (U/L)	38–174	-	325	371	907	-	127	76
C-Reactive protein (U/L)	0–3	-	-	-	-	16.7	96	-
Culture (Endotracheal Secretion)	n/a	-	-	-	-	^***^	-	-
^*^Film Array PCR Respiratory Panel	n/a	^**^	-	-	-	-	-	-

*Color code: uncolored boxes, values within the reference range; green boxes, values below the reference range; red boxes, values above the reference range; gray boxes, values not measured*.

*ICU, Intensive care unit*.

A nasopharyngeal swab specimen was tested for a respiratory panel of human pathogens and illness by

* *Film Array polymerase chain reaction (BioFire; Salt Lake City, UT, USA), including various viruses (Coronavirus 229E, Coronavirus HKU1, Coronavirus OC43, Coronavirus NL63, Adenovirus, Human Metapneumovirus, Human Rhinovirus/Enterovirus, Influenza A, Influenza A/H1, Flu-A-H1 2009, Flu-A-H1 PAN, Flu-A-H3, Flu-A-PAN-1, Flu-A-PAN-2, Influenza B, Parainfluenza virus 1, Parainfluenza virus 2, Parainfluenza virus 3, Parainfluenza virus 4, and VRS) and bacteria (Bordetella pertussis, Chlamydophila pneumoniae, and Mycoplasma pneumonia)*.

** Not detected;

*** *Acinetobacter baumannii complex associated health care infection*.

Given the many tropical infectious diseases with similar symptomologies to COVID-19 in Latin America, the medical staff thought the patient's presentation was most likely caused by a local infection. Also, since no confirmed case of COVID-19 had been reported in Panama at the time when the patient was admitted to the hospital, and the World Health Organization (WHO) had not yet declared the pandemic, he was not tested for SARS-CoV-2 using reverse-transcription-PCR (RT-PCR). Instead, the medical staff suspected that he had contracted the Hantavirus (HTV) or Sin Nombre virus of the *Bunyaviridae* family, an endemic and rarely fatal infection that causes severe pulmonary and renal complications in humans (12). Indeed, he had been in the Azuero peninsula visiting relatives during a family gathering 3 weeks earlier, which is the area with the most number of HTV cases per year recorded in Panama. He could have potentially been exposed to urine secretions from the animal reservoir (e.g., *Oligoryzomys fulvescens*) (12). However, the result for HTV test was negative. After receiving information from MINSA about the first confirmed COVID-19 case in the country, which was after 6 days of being hospitalized and more than 13 days of symptom onset, the patient underwent RT-PCR and tested positive for SARS-CoV-2 RNA. On March 6, 3 days before the SARS-CoV-2 infection was confirmed in the patient, his respiratory pattern worsened together with deterioration of the oxygenation parameters, requiring intubation and invasive mechanical ventilation. He was transferred to the ICU on March 9 (Figure 1; Table 1), where the medical team suspended treatment with steroids and began treatment with hydroxychloroquine, azithromycin, and lopinavir/ritonavir regimen according to WHO recommendations (4).

An epidemiological investigation began almost immediately to determine all contacts and potentially newly infected people. Approximately 15 people, including family members, students, friends, and colleagues were tested for SARS-CoV-2, none of whom tested positive or became ill with COVID-19. In addition, ~200 people from the school who had been in close contact with the director and the patient were followed-up clinically, and 10% were tested for SARS-CoV-2. The patient still does not know where he may have acquired the virus, as he had not been in close contact with the director or co-worker during the school organization week (Figure 1). Instead, cluster transmission might have been already occurring at the school, as seven more professors tested positive for SARS-CoV-2, after this patient was tested for the virus. Interestingly, none of these people or their relatives had traveled outside Panama in the last 12 months, and none developed severe symptoms or died due to COVID-19. As part of the first officially reported cluster of COVID-19 cases in the country, this case report served as evidence for health authorities to close the patient's school on March 10, 2020 (13), and shutdown the national education system just a day after (14).

Due to recurrent fever and neutrophilia, blood cultures and respiratory samples were obtained from the patient on March 18 (day 16 of hospitalization), showing an *Acinetobacter baumannii* complex infection without organ dysfunction, which was treated successfully (Figure 1). On March 19, the patient was extubated, weaned off the invasive mechanical ventilation, and discharged from the ICU after 4 days. He was officially designated as the first Panamanian resident to have recovered from an aggravated case of COVID-19 disease in the country (15). The patient's co-worker and director of the high school died on March 8, 2020, 5 days after being hospitalized. The autopsy revealed a prior infection with SARS-CoV-2 and resultant death due to COVID-19.

## Discussion

Panama was one the of the first Latin America countries to enter the list of territories affected by the COVID-19 pandemic. It has the largest testing rate per inhabitant in the region, and consequently the highest incidence of COVID-19 with 71,418 confirmed cases and 1,574 deceased by day 151 since the initial invasion (8). This makes it an ideal location to outline the potential epidemiological scenarios that might be present in the early stages of SARS-CoV-2 invasion in other countries of this region. Here, we described the epidemiologic chronicle of the first COVID-19 recovery case from Panama. The patient was a 49-year-old male high school teacher, who did not show any of the primary COVID-19 symptoms initially (i.e., fever, dry cough, and respiratory distress) as described by health authorities for seeking medical attention (16, 17). Nonetheless, he became severely ill over the course of 2 weeks and almost lost the battle against COVID-19.

Despite having severe pneumonia and being critically ill for 17 days, the medical staff claimed that he recovered quickly from COVID-19 because he did not have any underlying conditions such as hypertension, diabetes, and cardiovascular or renal diseases (17). From a clinical stand point, it is noteworthy to mention that the 4-days course of continuous infusion of steroids seems to have had a positive impact on the clinical condition of the patient, evidenced by the significant radiological improvement from March 9 to 20 (Figures 2D–G). However, steroid treatment in COVID-19 patients has been controversial, with inconsistent clinical outcomes (4, 16, 17). Similar to other COVID-19 case studies, the patient showed signs of lymphopenia and 16% increased levels of aspartate amino transferase (17–19). Further laboratory testing confirmed lymphopenia and elevated values of AST, ALT, C-reactive (CRP), and LDH levels. Nevertheless, leukocytosis was detected in the patient upon arrival at the hospital and remained until day 18 of hospitalization. This finding differed from previous publications where up to 31% of COVID-19 patients showed consistent signs of leukopenia (18–20). On day 18, his leukocyte count was 25.5 × 10^3^ cells/μl, due to an infection with the gram-negative bacterium *Acinetobacter baumannii* complex, which is one of the most frequent opportunistic pathogens causing hospital-acquired infections worldwide.

## Outbreak Investigation

Based on the case history, it appears that the patient was infected with SARS-CoV-2 before the “Carnivals” (*Carnavales* in Spanish), as he began feeling ill around February 21 and sought medical attention for the first time on February 24. The *Carnavales* started on February 22 and lasted for 4 days until February 26, 2020 (Figure 1) (21). *Carnavales* is one of the most popular holiday celebrations of the year in Panama, where thousands of locals and foreigners travel throughout the country to celebrate in social spaces or visit their family homes in the countryside. Whether or not the arrival and spread of SARS-CoV-2 in Panama can be associated with *Carnavales* remains to be answered, but such movements and gatherings could have facilitated numerous close contacts with potentially infected people over a short period of time and across long distances (17). The initial transmission of SARS-CoV-2 in Panama might have occurred during the preparation week (February 17–20), when 206 professors and 60 administrative workers shared the school with the first cluster of COVID-19 cases in the country, including our case in point. Some of these workers might have traveled overseas during late January and early February of 2020 for their annual vacation. Closing the school on March 10, 2020 a week after the beginning of the academic year, and immediately after becoming the first cluster of COVID-19 cases in the country, helped to reduce exposure to the virus and stopped further transmission among 4,200 teenaged students. This is corroborated by the lack of symptoms or deaths among the students from that school until now. To the best of our knowledge, there has not been any report published in the literature about the effectiveness of school closure in stopping the spread of SARS-CoV-2 infection to other geographic regions. In fact, this is currently a subject of much debate, and there is not much evidence against or in favor of this containment strategy. Our case report stands as an early circumstantial evidence that school closure could in fact be beneficial to inhibit the spread of SARS-CoV-2 to a larger portion of the academic community.

Panamanian health authorities must be on the lookout for early symptoms, including appetite loss, anosmia, and dysgeusia, and use them as sentinel signs of COVID-19 disease in order to treat people in a timely and effective manner (22–24). This is especially important to avoid further fatalities in individuals from the high-risk group, including those over 65 years of age, smokers, and/or those with co-morbidities (i.e., hypertension, diabetes, and cardiovascular or renal diseases). These symptoms can also help to differentiate between COVID-19 disease and other sign-related illnesses such as dengue, influenza, or HTV, especially now that some of these diseases are likely to emerge with the beginning of the rainy season in Panama. Dengue virus and HTV are just two examples of many other tropical diseases that are not observed in the northern hemisphere (e.g., Europe and the US), which were initially suspected as the cause of the disease in this patient. Therefore, not considering the diversity of zoonotic tropical pathogens that can cause misdiagnosis of COVID-19 could be a problem in the future as this emergent disease is likely to become the next neglected infection in the poor communities of tropical countries. Our report implies that SARS-CoV-2 must be included in the panel of respiratory infectious diseases to routinely test for potential COVID-19 cases, especially in the post-pandemic era.

This report suggests that patients who do not experience aggressive coughing and/or high fever in the early stages of infection might not spread the virus actively. Therefore, the use of face masks in public spaces along with actions of self-quarantine and social distancing are highly recommended to disrupt further transmission. Primary and secondary schools as well as universities must remain closed in Panama until sufficient herd immunity has been acquired in the susceptible population, and COVID-19 asymptomatic carriers no longer seem to be a threat to the local healthcare system. Homeschooling and online teaching could ultimately prevent waves of SARS-CoV-2 infections in the future due to reduced transmission rates.

## Limitations

We know that this is in fact the first COVID-19 patient who recovered from an episode of aggravated respiratory illness; therefore, he might have been closely associated with the first infected person who came to the school. A possible contact between our patient and a pre-symptomatic COVID-19 person cannot be ruled out, especially because this phenomenon has been reported in the early stages of an outbreak (25). However, due to the lack of epidemiological information from the other seven COVID-19 positive patients of the school, we cannot corroborate this possibility, nor can we discuss additional transmission scenarios among all these patients. After closure of the school by health authorities on March 10, 2020, 12 more people, mostly middle-aged adults, reported respiratory problems. However, confirmatory RT-PCR testing was not performed for these patients; hence, it is unknown if they were infected with SARS-CoV-2.

## Conclusion

The patient described in this case report was among the first patients admitted to the ICU in a public hospital in Latin America. Furthermore, he was among the first survivors, notwithstanding the fact that the doctors did not treat him with therapeutic practices and medication specifically targeting a SARS-CoV-2 infection. Our patient was treated for atypical pneumonia after 4 days of being hospitalized and more than 13 days of symptom onset. Surviving an infection with a new and deadly pathogen is a remarkable and fascinating clinical outcome considering the limited knowledge and preparation that the Panamanian medical staff had at the beginning of this invasion. The closure of a secondary school in Panama City due to the identification of the first cluster of SARS-CoV-2 activity, triggered the immediate shutdown of the education system in the entire country, which may have prevented the spread and slowed the transmission rate of COVID-19 during the early stages of invasion.

## Data Availability Statement

All datasets generated for this study are included in the article/supplementary material.

## Ethics Statement

Written informed consent was obtained from the individual(s) for the publication of any potentially identifiable images or data included in this article.

## Author Contributions

AH, PM, JR, and JL wrote the first draft of the manuscript with contributions from GE, JS, KR, and RG. GE, AH, PM, JS, and KR evaluated and analyzed the clinical history and laboratory testing of the patient. AH and PM treated the patient at the hospital. All authors read and approved the final manuscript.

## Conflict of Interest

The authors declare that the research was conducted in the absence of any commercial or financial relationships that could be construed as a potential conflict of interest.
